# Preliminary Observations of Personalized Repetitive Magnetic Stimulation (PrTMS) Guided by EEG Spectra for Concussion

**DOI:** 10.3390/brainsci13081179

**Published:** 2023-08-09

**Authors:** Milan T. Makale, Chad Nybo, Jason Keifer, Kenneth Blum, Catherine A. Dennen, David Baron, Keerthy Sunder, Igor Elman, Miles R. Makale, Panayotis K. Thanos, Kevin T. Murphy

**Affiliations:** 1 Department of Radiation Medicine and Applied Sciences, University of California San Diego, La Jolla, CA 92093, USA; 2 CrossTx Inc., Bozeman, MT 59715, USA; 3 Brain Health Hawaii, Honolulu, HI 96816, USA; 4 Department of Clinical Psychology and Addiction, Institute of Psychology, Faculty of Education and Psychology, Eötvös Loránd University, 1075 Budapest, Hungary; 5 Department of Psychiatry, Wright University, Boonshoft School of Medicine, Dayton, OH 45324, USA; 6 Department of Molecular Biology and Adelson School of Medicine, Ariel University, Ariel 40700, Israel; 7 Division of Addiction Research & Education, Center for Sports, Exercise & Global Mental Health, Western University Health Sciences, Pomona, CA 91766, USA; 8 Department of Family Medicine, Jefferson Health NE, Philadelphia, PA 19107, USA; 9 School of Medicine, University of California Riverside, Riverside, CA 92521, USA; 10 Cambridge Health Alliance, Harvard Medical School, Cambridge, MA 02143, USA; 11 Department of Psychology, University of California San Diego, La Jolla, CA 92093, USA; 12 Behavioral Neuropharmacology and Neuroimaging Laboratory on Addictions, Clinical Research Institute on Addictions, Department of Pharmacology and Toxicology, Jacobs School of Medicine and Biosciences, State University of New York at Buffalo, Buffalo, NY 14203, USA; 13 Department of Psychology, State University of New York at Buffalo, Buffalo, NY 14203, USA; 14 PeakLogic Inc., Del Mar, CA 92130, USA

**Keywords:** repetitive transcranial magnetic stimulation (rTMS), concussion, electroencephalogram (EEG), power spectrum, Rivermead Post Concussion Symptoms Questionnaire (RPQ), regression

## Abstract

There are no FDA-approved treatments for the chronic sequelae of concussion. Repetitive magnetic transcranial stimulation (rTMS) has been explored as a therapy but outcomes have been inconsistent. To address this we developed a personalized rTMS (PrTMS) protocol involving continual rTMS stimulus frequency adjustment and progressive activation of multiple cortical sites, guided by spectral electroencephalogram (EEG)-based analyses and psychological questionnaires. We acquired pilot clinical data for 185 symptomatic brain concussion patients who underwent the PrTMS protocol over an approximate 6 week period. The PrTMS protocol used a proprietary EEG spectral frequency algorithm to define an initial stimulation frequency based on an anteriorly graded projection of the measured occipital alpha center peak, which was then used to interpolate and adjust regional stimulation frequency according to weekly EEG spectral acquisitions. PrTMS improved concussion indices and normalized the cortical alpha band center frequency and peak EEG amplitude. This potentially reflected changed neurotransmitter, cognitive, and perceptual status. PrTMS may be a promising treatment choice for patients with persistent concussion symptoms. This clinical observational study was limited in that there was no control group and a number of variables were not recorded, such as time since injury and levels of depression. While the present observations are indeed preliminary and cursory, they may suggest further prospective research on PrTMS in concussion, and exploration of the spectral EEG as a concussion biomarker, with the ultimate goals of confirmation and determining optimal PrTMS treatment parameters.

## 1. Introduction

Mild traumatic brain injury (mTBI) is generally referred to as concussion, and is chiefly caused by motor vehicle accidents, falls, assaults, and sports-related impacts [1,2]. Concussion is widespread and in a significant proportion of affected patients it is disabling [2,3,4,5]. There are few treatment options, and the long-held perspective that mild concussion resolves relatively rapidly and without sequelae is being questioned [6,7,8]. Several reports have related higher rates of depression and dementia with a history of concussion [9,10,11]. The need for effective treatments has prompted the use of repetitive transcranial magnetic stimulation (rTMS), which has shown beneficial activity in concussion, although results have been mixed [2]. We have developed a comparatively dynamic form of rTMS, called personalized rTMS (PrTMS), which methodologically aligns with emerging mechanistic data on the pathophysiology of concussion [12,13]. Based on the previous application of standard (non-personalized) rTMS for mild traumatic brain injury, despite mixed results, we decided to perform this study utilizing our personalized approach in a large number of mTBI patients (n = 185) with persistent concussion symptoms. 

A recent review by Mollica and co-workers (2021) showed that rTMS concussion studies have been small in scope, ranging between 6 and 29 subjects [2], and clearly much remains to be learned about the neurophysiology and neurobiology of concussion. Nonetheless, the ability of rTMS to entrain and synchronize neurons has attracted attention for the re-establishment of normal, distributed alpha oscillatory rhythms. RTMS involves an external scalp-level induction coil that, in response to a strong, rapidly pulsed high-voltage electrical current (kiloVolts (kV), and kiloAmps (kA)), generates a pulsed magnetic field, which in turn induces within the electrical environment of the brain cortex a pulsed electrical field. This field entrains the firing frequency of cortical neurons in the alpha band, which is the dominant frequency band in the awake, conscious human brain [2]. 

Importantly, inflammatory activity may be reduced by rTMS, along with reactivatation of neurotransmitters to rebalance signaling, reduce depression symptoms, and set in motion neuroplasticity for CNS repair [14,15,16,17]. Koski et al. (2015) found in a non-sham controlled study that rTMS substantially erduced symptoms according to the post-concussion symptoms scale (*p* = 0.009), but some subjects had increased headache and sleep disturbances [18]. Meek and colleagues (2020) conducted a non-sham controlled pilot study of rTMS in concussion and found significant improvements in symptoms [19]. Siddiqui et al. (2019) examined rTMS for concussion but found no improvement in cognition [8]. Moussavi et al. (2019) performed a sham controlled study in which the primary outcome was the Rivermead Post Concussion Symptom Questionnaire (RPQ), and found no significant difference between sham and active rTMS [20]. The review by Mollica et al. (2021) was a meta-analysis of 342 studies of rTMS in mTBI [2]. The authors concluded that in sham-controlled studies, 1 to 4 weeks of rTMS showed benefits for post-concussive headache and depression, but, importantly, not all studies showed patient improvement. Oberman et al. (2020) published a scoping review of military populations treated by rTMS for concussion and concluded that support for the efficacy of rTMS in concussion is limited [21]. 

Clearly, the results for rTMS in concussion, while promising, are mixed, and there are two key considerations that may highlight the applicability of our modified form of rTMS, called PrTMS, for concussion treatment. First, our experience with the spectral EEG in many concussion patients has shown frequent diffuse cortical frequency irregularities, extending from the orbital frontal cortex posterior to the visual cortices. Others have also noted a large variability and distribution of EEG brain wave frequencies in concussion [12]. Individualized, i.e., personalized, rTMS treatment regimens have been proposed based on the large parameter space presented by rTMS [8]. We hypothesized that treatment delivery to all or most dysregulated cortical locations would be necessary to re-establish oscillatory synchrony. Standard rTMS is unable to accomplish this, due to relatively high treatment intensity (amplitude), which is at, or above, the motor neuron threshold. The direct treatment of the motor-sensory strip, for example, would cause seizure. 

Low-amplitude stimulation with PrTMS allows the direct stimulation of the motor-sensory strip and other sensitive cortical locations, with a much diminished risk of overstimulation causing seizure. There is a developing literature and awareness that lower levels of stimulation do activate neurons, as described by Moretti et al., 2022, and Zmeykina et al., 2020 [22,23]. Standard rTMS also typically involves the delivery of a fixed, one-size-fits-all, 10.0 Hz treatment frequency. Based on the literature, we believe that in many patients, 10.0 Hz may not be the right frequency, and may be a factor responsible for a lack of response to rTMS in some patients, as noted by Leuchter et al., 2021, Klooster et al., 2022, and Gogulski et al., 2023 [24,25,26,27]. There is recent evidence that the frequency of alpha oscillatory activity allows synchrony and coordinated activity between brain regions (Figueira et al., 2020) [28]. The alpha amplitude peak center frequency varies between individuals, and therefore the proposed rationale is that rTMS should entrain brain regions at each subject’s specific alpha center frequency. Deviating from this intrinsic frequency may result in an inadequate resetting of corticothalamic oscillators and suboptimal communication between cortical territories, as described by Roelofs et al., 2021, Jin et al., 2006, Garnaat et al., 2021, and Leuchter et al., 2015 [24,29,30,31]. In fact, in 2006, Jin and colleagues reported that using individualized alpha center peak stimulation frequency provided superior outcomes in schizophrenic patients [30]. 

Additionally, the reliability of traditional cognitive assessment tools and imaging has increasingly been questioned, and there is an expanding focus on non-subjective assessments that are based on the spectral EEG to evaluate post-concussive brain alterations that are otherwise difficult to identify [32]. 

Based on the foregoing, PrTMS may offer a solution, as spectral EEG analyses in addition to psychological self-reported questionnaires are used to guide frequent adjustments of stimulus frequency, along with the identification and treatment of multiple affected cortical sites. The alpha oscillatory activity of the brain cortex has been addressed via the EEG 1/f^a^ power spectrum, an analytical approach initially described in detail by Voytek et al. (2015), that aligns with early publications addressing the importance of synchronous brain oscillations, and that has since expanded [33,34,35,36]. Changes in the 1/f^a^ aperiodic component within each individual may reflect shifts in dominant cortical neurotransmitters as well as changed cognitive status and perceptual encoding, and could advance as an independent biomarker of neuropsychiatric disorders [33,37]. 

Here we report preliminary observational clinical outcome data obtained with mTBI patients who had persistent symptoms of concussion and were seeking effective treatment options. These patients likely had variable periods of persistent symptoms, but none had new concussion. This was a treatment program, not a prospective study, and a number of variables were not recorded such as time since injury and levels of depression. We treated these individuals with PrTMS, and our data, which were preliminary, may imply that PrTMS was associated with an improvement in symptoms. We present EEG alpha band center frequency and 1/f^a^ spectral power spectrum analyses for concussion patients, which exhibited changes after PrTMS therapy. Despite the difficulty and rarity of incorporating control groups in concussion studies, the outcomes and their EEG correlates presented here may suggest more comprehensive sham controlled prospective studies of PrTMS versus rTMS and standard therapy alone for concussion. Importantly, 1/f^a^-based analyses of the spectral EEG may serve as a much needed and readily acquired biomarker in concussion. 

## 2. Methods

### 2.1. Subjects

Males and females came to our clinic indicating that they had persistent concussion symptoms (male/female ratio = 1.4:1) and were screened for concussion using either the self-reported Median Concussion Symptom Inventory (CSI) or the Rivermead Post Concussion Symptoms Questionnaire (RPQ). The patients were of all ethnicities, the average age was 38 years, and 185 patients received 6 weeks of PrTMS treatment. Patients and/or their families sought out our clinic because of persistent concussion symptoms and the desire for an effective treatment option. The rTMS eligibility criteria defined by Rossi et al. (2009), McClintock et al. (2018), and Rossi et al. (2021) were used for patient screening [38,39,40]. Patients were briefed on the treatment procedures and they provided informed medical consent for PrTMS. Moreover, before our retrospective review, institutional review board (IRB) approval was obtained: WCG IRB Study number 1254094; IRB tracking number 20190239; Title: A Retrospective Review of Personalized Repetitive Magnetic Stimulation (PrTMS). Patients were encouraged to continue their standard psychotherapy and/or medication management during the course of PrTMS treatment. The duration of treatment was open-ended and was predicated on patient preferences in the context of perceived and quantifiable progress.

### 2.2. Treatment Schedule

**Figure 1:**
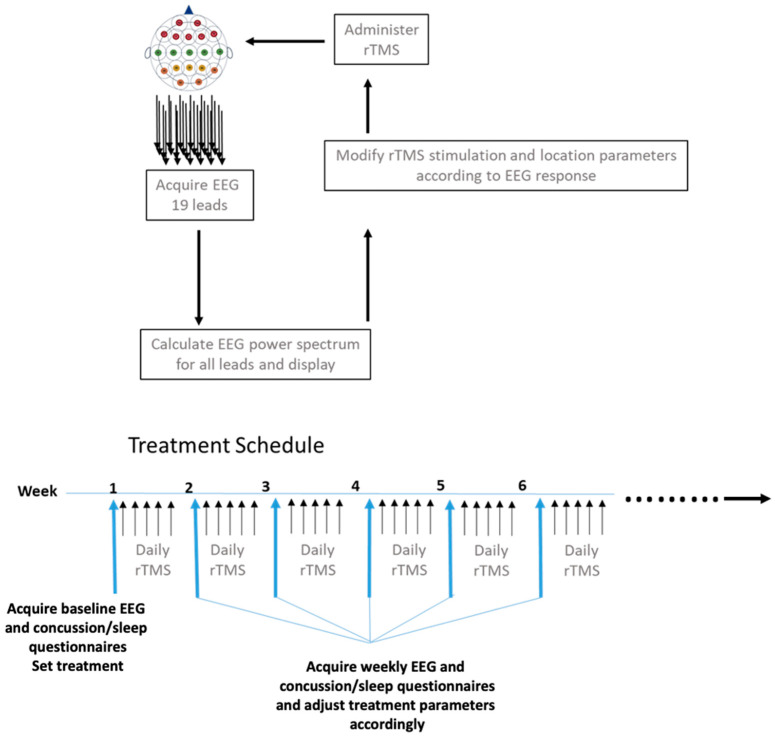
PrTMS treatment plan and schedule. RTMS for personalized administration (PrTMS) was adjusted weekly in terms of stimulation amplitude, frequency, intertrain interval, length of treatment train, and cortical locations (a minimum of 3 and maximum of 5 locations) treated each day. The EEG was acquired weekly and analyzed spectrally, and concussion questionnaires and sleep questionnaires were also administered every week.

PrTMS was administered once daily for five days a week as shown in Figure 1, and the duration of treatment was typically 6–8 weeks or 30–40 treatments. After experiencing improvements at 6 weeks, many patients discontinued treatment, and here we compare data for 6 weeks versus pretreatment, since we had the same number (n = 185), and other time points. Importantly, an electroencephalogram (EEG) was acquired regularly for each patient, as this neurophysiological measure represents an independent, non-subjective treatment response indicator [32]. Hence, the EEG was obtained before PrTMS commenced, and on the first day of each week of PrTMS. Power spectrum analyses of all 19 leads and a single heart lead were then rendered into a display, and plotted in time series along the “x” axis from 2 to 20 Hz. A proprietary frequency algorithm PeakLogic Inc., San Diego, CA, USA) defined an initial stimulation frequency, which was a result of a mathematical summary of the aggregate alpha center peak frequency, minus the “noise factor” introduced into the rendering from the destructive wave interference(s) created by all other measured waves. This algorithm was used to interpolate and adjust regional stimulation frequency, amplitude, length of train, intertrain interval, and number of treatments according to weekly EEG spectral acquisitions. In addition, concussion symptoms and sleep quality in a small subset of patients were assessed weekly using the self-report Concussion Symptom Inventory (CSI), the Rivermead Concussion Symptom Inventory (RPQ), and the Sleep Condition Indicator (SCI) questionnaires, respectively. Patients had follow-up questionnaire and EEG visits at 11 weeks and a few patients (n = 13) returned at 24 weeks. Activities, sleep patterns, sports participation, etc., were not monitored after the patients finished their 6-week course of PrTMS.

### 2.3. EEG Data Acquisition

EEG recordings were acquired before PrTMS and just before every sixth treatment as long as PrTMS continued. The EEG was recorded from awake, eyes closed, seated subjects using a 19-lead high impedance dry electrode EEG headset (Cognionics (CGX) Inc., San Diego, CA, USA). Data filtering was avoided and technically flawed recordings were removed by an experienced observer. A four-minute EEG time epoch was transformed via Welch’s Fast Fourier Transform (FFT) using a custom Python program, to produce a 2–20 Hz power spectrum with 0.1 Hz resolution. 

### 2.4. Personalized Repetitive Transcranial Magnetic Stimulation (PrTMS)

PrTMS was delivered by a qualified, trained rTMS technician using a MagVenture MagPro R30 transcranial stimulator and B-65 head transducer. Patients were seated in a quiet room with the eyes closed and without sedation. Magnetic field intensity was gradually increased over the course of treatment. Stimulation intensity was 25–60% of the typical resting motor threshold (MT), and the stimulus frequency range was 8–13 Hz, with magnetic pulses delivered in 10–15 s trains. Intertrain intervals began at 30 s, and gradually decreased to 10 s. In order to avoid overstimulating the cortex, we initiated treatment with low power levels and then increased until a response was observed based on the spectral EEG and the concussion symptom questionnaire. Experience with our proprietary algorithm with previous neuropsychiatric disorders indicated that this procedure might be helpful in promoting a change in firing frequency for each site. During each treatment session, the motor-sensory strip and subsequent prefrontal and frontal regions were treated in succession. Clinical personnel evaluated patients daily for adverse events (AEs) including headache, scalp pain, cognitive deficits, seizures, observed or volunteered problems, complaints, physical signs and symptoms, medical conditions that were not previously present, and previous medical conditions that worsened.

### 2.5. Data Analysis and Statistical Methods

#### 2.5.1. Rivermead and Sleep Quality Scores

The primary study endpoint was the reduction in symptoms measured by concussion questionnaires, including the Concussion Symptom Inventory (CSI) and the Rivermead Concussion Index (RPQ), and the sleep quality questionnaire (Sleep Condition Indicator—SCI). These were the only continuous variables, acquired weekly from baseline (pretreatment) to final treatment. The median/mean change from baseline (CFB) data were tested with α = 0.05 level of significance. There was no adjustment for multiplicity, and missing data imputation was not implemented. 

#### 2.5.2. EEG Spectral Analyses

The dominant alpha peak (center) frequency was determined for all EEG leads, averaged for each cortical region, and the amplitude of the alpha band (8–13 Hz) spectral center frequency was identified in each EEG lead for each week of treatment. The 1/f^α^ aperiodic spectral component was determined by averaging the 2–20 Hz power spectrum amplitude from the 7 leads in the frontal cortex, plotting log power versus log frequency, and then calculating the robust regression line and its slope, treating periodic oscillatory components as outliers. The amplitude of the alpha (slope) from zero was then determined. 

## 3. Results 

### 3.1. Concussion Symptom Inventory (CSI)

**Figure 2:**
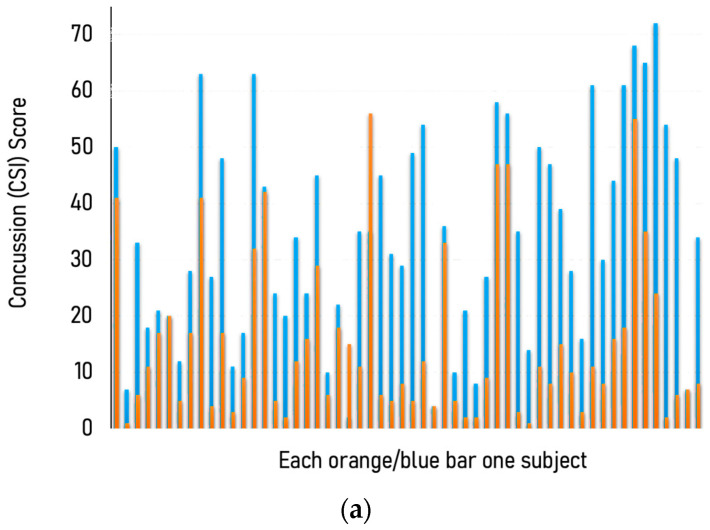

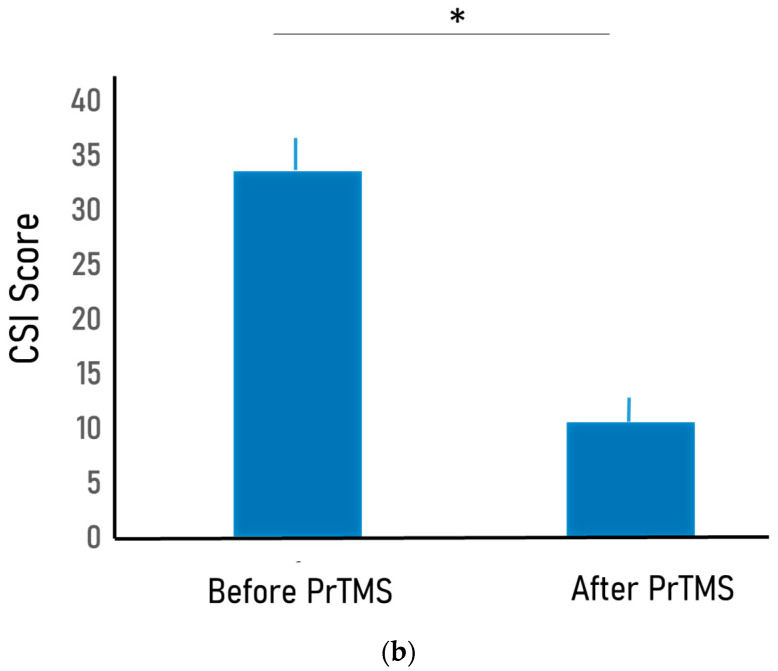
(**a**) Consistent reduction in concussion symptoms inventory (CSI) across a cohort of 56 individual patients. Each orange and blue combined bar is one subject, and the blue portion indicates the CSI before PrTMS, while the orange segment denotes the CSI score after PrTMS. A paired 2-tailed parametric *t*-test compared before vs. after scores (*p* < 0.0001). The average number of treatments was 9 and the range was 6 to 19. (**b**) Median concussion symptom inventory (CSI) in a cohort of 56 patients of all ages before and after PrTMS. Average number of treatments is 9 and the range was 6 to19. Mean before and after PrTMS CSI scores are shown for all 56 patients and SEMS are indicated on the bars. A parametric, 2-tailed paired t-test compared before versus after PrTMS for all subjects (* *p* < 0.0001).

Importantly, while this paper reports the outcomes obtained from medical treatment, and is not based on a prospective study, the patients all had persistent concussion symptoms for which they sought a viable treatment option. The Concussion Symptom Inventory (CSI) in 56 patients of all ages detected a significant decline in concussion symptoms after PrTMS was initiated, as shown in Figure 2a,b, from a mean of 33.5 to 10.5 (paired *t*-test, *p* < 0.0001). The mean number of treatment days in this group was 9 and ranged between 6 and 19, suggesting that patients responded rapidly, and that they were indeed responding to treatment. Only 2 of 56 patients failed to respond. While a distinct cutoff score has not been defined for the CSI, patients exhibited marked improvement with mean scores dropping by almost 70%.

### 3.2. Rivermead Concussion Questionnaire (RPQ) Scores

**Figure 3:**
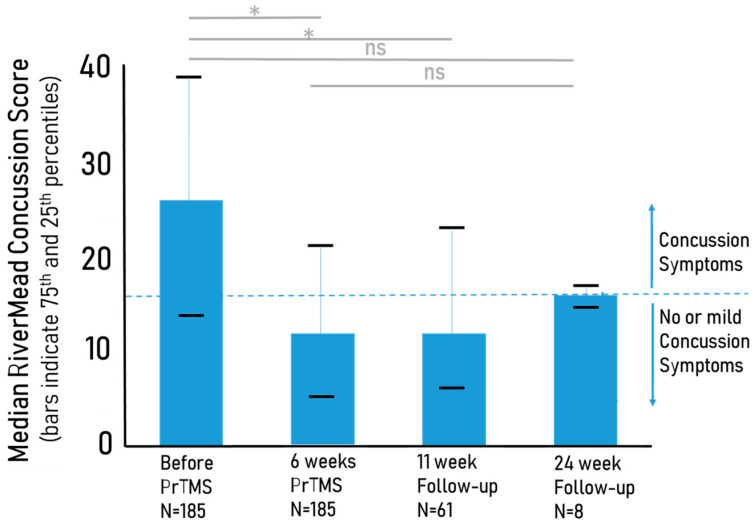
Reduction in median Rivermead Concussion Inventory (RPQ) scores for 185 patients of all ages suffering from persistent concussion symptoms before and after PrTMS. The blue bars show the median concussion score before treatment, at 6 weeks of treatment, and at the 11- and 24-week follow-ups. The horizontal black bars indicate the 75th and 25th percentiles for each time point, and the putative and approximate score threshold of 16, which roughly divides concussion versus no concussion, is denoted by the dashed line, while ns indicates ‘not significant’. All of the after-PrTMS scores indicate very mild or no concussion symptoms (highly significant * *p* < 0.01 for pretreatment versus weeks 6 and 11, repeated measures ANOVA). Note that at the 24-week follow-up the median score was lower than pretreatment and higher than at 6 and 11 weeks, but was not statistically significantly different at the *p* < 0.05 level from pre-treatment or from 6 and 11 weeks, likely due to the small sample size (n = 8).

Initially, the median Rivermead concussion questionnaire (RPQ) score for 185 patients was 26, which is above the threshold of 16 for concussion according to Thompson et al. (2020). After 6 weeks of treatment the mean score was reduced to 12 (n = 185), and at the 11- and 24-week follow-ups the mean scores were 12 (n = 61) and 16 (n = 8), respectively, as depicted in Figure 3. A repeated measures ANOVA, applied sequentially with the maximum Bonferroni correction because of the declining sample size due to attrition by 11 weeks and after, indicated that these pre- versus post-treatment differences from baseline to 6 weeks and the 11-week (*p* < 0.05) follow-up were significant. It should be noted in this context that while the total Rivermead score indicates the severity of the post-concussive syndrome, a broadly accepted definitive clinical cut-off score has not been established. Some sources have suggested an approximate threshold of 16 for clinically significant concussion symptoms, according to Creyos Health (https://creyos.com/resources/articles/measure-concussion-effects-with-the-rpq, accessed on 5 August 2023) and work reported in abstract form by Thompson and co-workers (Brain Injury 2016 30(5–6):770). Regardless, RPQ scores were clearly reduced after PrTMS. However, a follow-on sham controlled prospective study, optimally with a crossover design, is needed to determine whether PrTMS treatment effects exceed any placebo effect. 

### 3.3. Sleep Quality and Insomnia Scores 

**Figure 4:**
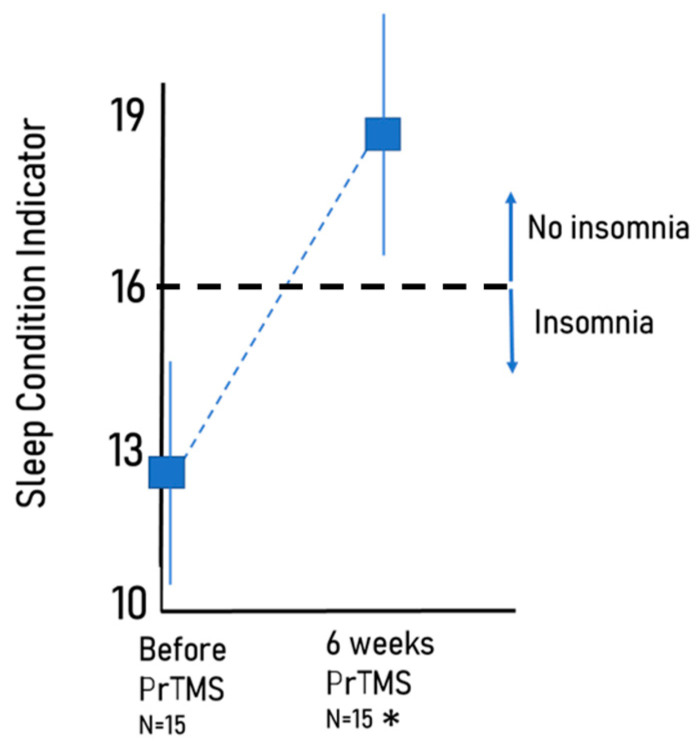
Average increase in the Sleep Condition Indicator (SCI) score. The average improvement was from 12.4 before treatment to 18.7 at 6 weeks (SEM shown, * *p* < 0.0034, paired *t*-test). When the SCI was less than or equal to 16, which is shown on the graph by the dashed line, the patient had probable insomnia. There were 15 subjects.

Patients treated with PrTMS reported at least some sleep improvement according to the self-reported Sleep Condition Indicator (SCI) (Figure 4). The mean sleep quality score improved (a higher score denotes improvement) from 12.4 to 18.7 after 6 weeks of PrTMS (*p* = 0.0034, n = 61). This post treatment value is above the putative CSI cut-off of 16, which, according to the definition of Espie et al. (2014), correctly identifies 89% of subjects as having probable insomnia disorder, and 82% of subjects as not having insomnia disorder [41]. 

### 3.4. EEG Alpha Bband Center Frequency and 1/f^a^ Spectral Regression

**Figure 5:**
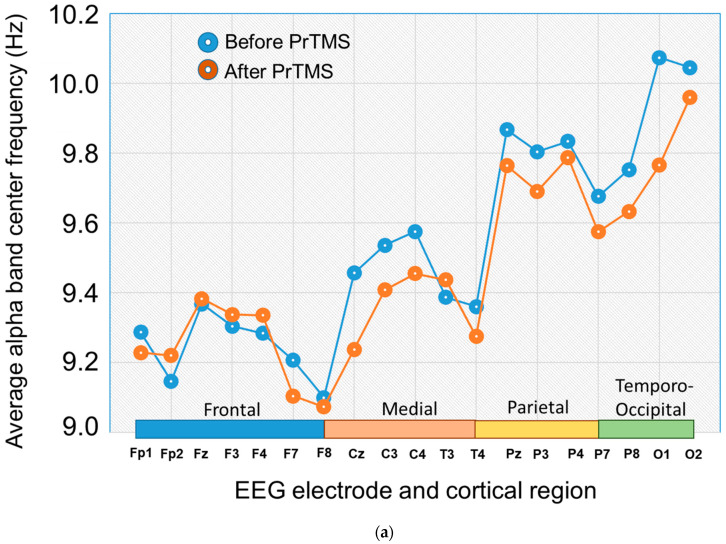

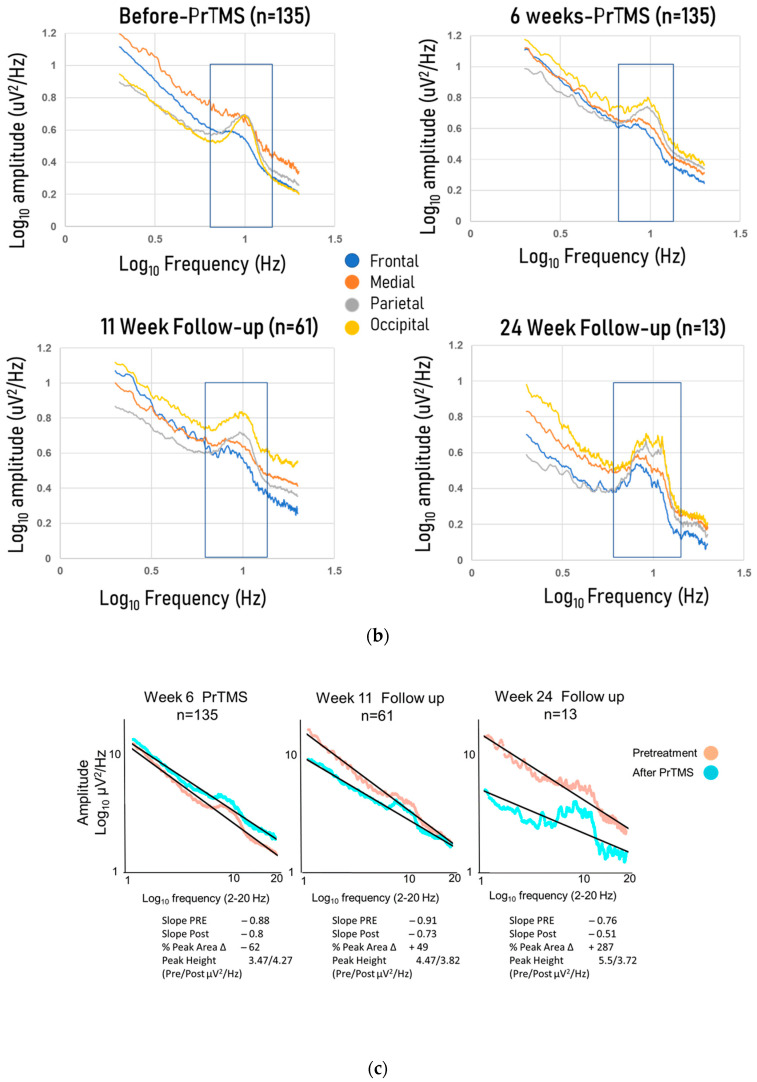
(**a**) Alpha peak center frequencies at 6 weeks after PrTMS. The alpha band peak center frequencies for all brain regions (BR1 = frontal, BR2 = central, BR3 = parietal, BR4 = occipital) in subjects suffering from persistent concussion symptoms. A repeated measures ANOVA indicated that the post-PrTMS alpha frequency change, although relatively small, was significant (*p* < 0.0035). (**b**) Logarithmic plots of averaged EEG power spectra for each brain region at each time point. The four panels show mean log–log plots of frontal, medial, parietal, and occipital EEG power spectra for all subjects suffering from persistent concussion symptoms. The time points are before PrTMS, at 6 weeks (n = 135) of PrTMS, and at the 11-week (n = 61) and 24-week (n = 13) follow-ups. Note the disorganized appearance of the power spectrum before PrTMS both outside and within the alpha peak (box outline). After 6 weeks of PrTMS the expected posterior–anterior amplitude gradient, i.e., occipital > parietal > central > frontal, for the alpha peak was re-established (highlighted by the box outline), and the brain region spectra exhibited close overall alignment. The 11- and 24-week follow-up spectra maintained the posterior–anterior gradient, but the amplitudes of the overall spectra diverged somewhat. A limitation of the 24-week data is that they were acquired with only 13 subjects. At pretreatment and at 6 weeks, 135 out of 185 subjects had high-quality EEG recordings that in a blinded way were deemed sufficient for proper analysis. (**c**) Frontal 1/f^a^ Regressions of EEG spectra. The 3 panels show mean log–log plots of frontal EEG power spectra for subjects before PrTMS, at 6 weeks of PrTMS, and at the 11-week and 24-week (n = 13) follow-ups. The solid lines in each panel indicate the 1/f^a^ robust regressions. The alpha peak center frequency signal amplitude is shown in µV^2^/Hz. The 1/f^a^ slope consistently exhibited a positive change in slope, i.e., shallower at 6, 11 and 24 weeks, all of which were found to be statistically significant using a paired *t*-test (*p* < 0.05).

The EEG potentially may provide a useful, non-subjective index of concussion patient status and response to treatment. While questionnaires may contain some subjective bias, neurophysiological measures such as the EEG are likely independent of the subject’s personal perceptions and are objective. We observed that at the sixth EEG, i.e., after 5 weeks of PrTMS, the alpha band center frequency declined for all brain regions, as shown in Figure 5a. A repeated measures ANOVA showed that the reduction for all brain regions together was significant (*p* = 0.0035).

The alpha peak portion of the EEG power spectrum for all four brain regions in the subjects assumed its expected relative amplitude and shape after 6 weeks of PrTMS (Figure 5b). By 6 weeks the alpha band spectra appear more synchronous, and at the 11- and 24-week follow-ups, they remain more synchronous than before treatment, but appear less aligned than at 6 weeks. At the 24-week follow-up, the EEG alpha peak amplitude was much reduced and the center frequency declined, although there were only 13 patients in this group. The relative area of the alpha peak compared to pretreatment was reduced at 6 weeks of PrTMS, while at the 11- and 24-week follow-ups, compared to respective pretreatment values, it was greater, as indicated in Figure 5c.

The 1/f^a^ regressions of averaged power spectra (Figure 5c) for the frontal cortical EEG leads are shown for before and after treatment. The regression lines have shallower slopes at 6 weeks of PrTMS and at the 11-week follow-up (*p* < 0.0001), and show reduced steepness relative to pretreatment at the 24-week follow-up (*p* < 0.0001). This suggests that the degree of arousal of the brain and its neurotransmitter profile may have changed with PrTMS or over time [33,36,37]. To our knowledge, this is the first report of spectral analysis applied to patients with concussion, and our results suggest the possibility that the EEG spectrum may potentially serve as a concussion biomarker and a means to track post-injury trajectory and recovery [42]. 

## 4. Discussion

Mild to moderate concussion is widespread; it is difficult to manage, and there are no US Food and Drug Administration (FDA)-approved specific medications for any neuropsychiatric or neurocognitive concussion symptoms [21]. The present report describes the use of a modified form of rTMS called PrTMS, with patients suffering from persistent concussion symptoms and who sought an effective treatment option. Two different concussion indices both indicated an improvement of symptoms with PrTMS. After 6 weeks of PrTMS there was a frontal cortical increase in the spectral EEG alpha peak amplitude, and an initial increase in the alpha peak center frequency. Central, parietal, and occipital cortical regions showed a decline in center frequency and a rise in alpha peak amplitude. 

The underlying mechanisms of concussion involve changes in neurotransmitter activity, and this points to the potential relevance of 1/f^a^ regression analysis of the spectral EEG [43]. Accordingly, we observed that 6 weeks of PrTMS induced a slight but statistically significant slope decrease, i.e., a shallower slope, of the 1/f^a^ regression of the frontal cortical spectral EEG, reported for the first time in concussion. This shallower spectral slope may reflect subtle changes in frontal cortical neurotransmitter balance, neural irregularity, and cognitive status and perceptual encoding [36,44]. Washke et al. (2017) and others noted that encoding and representing sensory information is more thorough during an irregular or desynchronized state as opposed to a regular, or synchronized, condition [44,45,46]. Not only is this of considerable interest as a potential biomarker in concussion, but may also suggest potential explorations of mechanisms and possible pharmacological strategies, conceivably in the context of a combined pharmacotherapy–PrTMS approach. 

Electrical stimulation and assessment of the brain for the treatment of persistent concussion symptoms has long been considered for the treatment of TBI, and has emerged in recent years via several different modalities including transcranial direct current stimulation (tDCS), magnetoencephalography (MEG), and rTMS. Rudroff and Workman, 2021, conducted a comprehensive review of the effects of tDCS in mTBI [47]. The authors found just three studies that examined tDCS for mTBI, and concluded that there is high intersubject variability in outcomes and limited evidence that tDCS has a significant beneficial effect in mTBI. Peitz and colleagues (2021) noted MEG is a functional brain imaging technique that has high temporal resolution and may provide information that is not provided by standard imaging methodologies [48]. They proposed that that MEG findings may correlate with post-concussive symptoms, which could eventually be clinically useful. Kundu et al. (2018) discussed the use of deep brain stimulation (DBS) for TBI [49]. This technique involves the surgical placement of electrodes in the brain and targets deep brain nuclei and white matter tracts with millimeter accuracy. Injury to the thalamic nuclei may play an important role in concussion symptoms, and these sites have become important targets in DBS. There is notably a paucity of literature on DBS for concussion, although Lee et al. (2013, 2015) found that the stimulation of the medial septal nucleus and the septohippocampal region in concussed rats increased hippocampal theta oscillations in TBI rats and resulted in improved cognitive performance [50,51]. DBS can provide stimulation of targets 24 h a day and can be customized for each patient, but there is always surgical risk, and a small (1%) risk of brain hemorrhage, headache, or worsening of symptoms. Ghaffarpasand et al. (2014) noted that DBS may be effective for severe TBI sequelae, although evidence is lacking, and there is the potential for serious complications [52]. Cunningham et al. (2016) indicated that DBS carries risk, in addition to surgical complications and sensory and motor side effects, which includes very significant mood disturbances and depression with suicidal urges [53]. 

External brain stimulation via electromagnetic fields has been explored as a safe, effective, and economical approach to treating TBI. In this context, rTMS is a leading candidate modality and many animal models support the use of rTMS for TBI. Yang et al. (2012) reported that 15 Hz electromagnetic fields were neuroprotective in rats with TBI [54]. In contrast, Yoon et al. (2015 did not find evidence of motor recovery from rTMS in a rat model of TBI [55]. However, Sasso and colleagues (2016) found that rTMS reduced degeneration and inflammation in a rat model of focal brain damage [16]. Sekar et al. (2019) reported that the low-field magnetic stimulation of deep cortical and subcortical areas in a mouse model of repeated traumatic brain injury restored cognitive and motor functions [56]. Several investigators including Lu et al., 2015, Lu et al. 2017, and Verdugo-Diaz et al., 2017 all tested rat TBI models with rTMS, and in aggregate, they observed beneficial effects of rTMS on recovery, performance, and histology [57,58,59]. 

Electroconvulsive therapy (ECT) has been reported by Cunningham et al. (2016) to be effective for post-TBI Parkinson’s disease, and has been established as an effective treatment option [53]. Interestingly, these authors observe that ECT may possibly exert its effects in Parkinsons Disease by increasing dopaminergic transmission in the striatum. RTMS modulates dopamine along with GABA, and concussion may affect GABAergic thalamic neurons and dopamine signaling [60,61,62]. Lan and co-workers (2019) suggested that dopamine should be considered a first line treatment in TBI [63]. Several neurotransmitter types and pathways may play key roles in the deficits associated with concussion, and may represent potential therapeutic targets. For example, reduced levels of the neurotransmitter GABA over one year following traumatic brain injury were measured by Kang et al. (2022) [64]. In the same study, longitudinal improvement in executive attention correlated with increased GABA receptor availability. Arakaki et al. (2018) suggested that cholinergic mechanisms may participate in the learning impairment seen after mTBI [65]. Along these lines, others similarly suggest that the dysregulation of consciousness induced by concussion could be due to enhanced acetylcholine as well as concomitant lowered norepinephrine in the cerebral cortex [66]. Disturbances in memory, focus, and problem-solving are common after mild to moderate TBI, which could reflect cholinergic dysfunction. Midline concussive injury in rats induced a bilateral loss of cholinergic neurons averaging 36% in area Ch1 (medial septal nucleus), 45% in Ch2 (nucleus of the diagonal band of Broca), and 41% in Ch4 (nucleus basalis of Meynart). In addition, lateralized injury induced cholinergic neuron loss of similar magnitude ipsilaterally, but a lower contralateral loss of between 11% and 28% [67].

Our preliminary study has several key limitations, most notably the absence of a sham controlled study cohort, cursory clinical observations, and the heterogeneous nature of the treated population. While there were no patients with “new” concussion, the time periods of persistent concussion symptoms were likely variable and were not reported. Since this was a treatment-based endeavor, we assessed patients for the presence of concussion and did not measure possible co-morbidities such as depression. Nonetheless, we suggest that the current findings in a moderately sized cohort (185 patients) imply a possible beneficial clinical outcome. This of course has to be rigorously validated, especially given the very large placebo effects of rTMS. A large prospective study, e.g., double blind–sham controlled incorporating a cross-over design, comparing standard rTMS to PrTMS, is needed to determine if PrTMS creates positive effects that extend beyond placebo. 

## 5. Conclusions

The present preliminary report summarizes pilot clinical data acquired with the PrTMS treatment of patients suffering from persistent symptoms of concussion. Patients reported substantial and significant improvement in self-reported concussion and sleep indices after PrTMS. Moreover, the spectral EEG, a comparatively agnostic measure of cortical status, changed in terms of alpha peak properties, apparent synchrony between cortical territories, and 1/f^a^ regression slope. In aggregate, these findings support the pursuit of further, prospective controlled studies of PrTMS for concussion treatment, along with exploration of the spectral EEG as a biomarker of concussion, and the examination of the persistence of comorbidities such as depression.

## Data Availability

The data presented in this study are available on request from the corresponding author. The data are not publicly available due to privacy concerns, all subject identifiers must be removed prior to our making the data available.
